# Temsavir Modulates HIV-1 Envelope Conformation by Decreasing Its Proteolytic Cleavage

**DOI:** 10.3390/v15051189

**Published:** 2023-05-18

**Authors:** Marianne Boutin, Halima Medjahed, Manon Nayrac, Rishikesh Lotke, Gabrielle Gendron-Lepage, Catherine Bourassa, Daniel Sauter, Jonathan Richard, Andrés Finzi

**Affiliations:** 1 Centre de Recherche du CHUM, Montreal, QC H2X 0A9, Canadamanonnayrac@gmail.com (M.N.);; 2 Département de Microbiologie, Infectiologie et Immunologie, Université de Montréal, Montreal, QC H3T 1J4, Canada; 3 Institute for Medical Virology and Epidemiology of Viral Diseases, University Hospital Tübingen, 72076 Tübingen, Germany; 4 Institute of Molecular Virology, Ulm University Medical Center, 89081 Ulm, Germany

**Keywords:** HIV-1, Env glycoprotein, entry inhibitors, fostemsavir (BMS-663068), temsavir (BMS-626529), proteolytic cleavage, bNAbs, ADCC

## Abstract

HIV-1 envelope glycoproteins (Envs) mediate viral entry and represent a target of choice for small molecule inhibitors. One of them, temsavir (BMS-626529) prevents the interaction of the host cell receptor CD4 with Env by binding the pocket under the β20–β21 loop of the Env subunit gp120. Along with its capacity to prevent viral entry, temsavir stabilizes Env in its “closed” conformation. We recently reported that temsavir affects glycosylation, proteolytic processing, and overall conformation of Env. Here, we extend these results to a panel of primary Envs and infectious molecular clones (IMCs), where we observe a heterogeneous impact on Env cleavage and conformation. Our results suggest that the effect of temsavir on Env conformation is associated with its capacity to decrease Env processing. Indeed, we found that the effect of temsavir on Env processing affects the recognition of HIV-1-infected cells by broadly neutralizing antibodies and correlates with their capacity to mediate antibody-dependent cellular cytotoxicity (ADCC).

## 1. Introduction

The human immunodeficiency virus type 1 (HIV-1) envelope glycoprotein (Env) is synthetized in the endoplasmic reticulum, where the gp160 precursor undergoes trimerization and the addition of glycans. During its transport to the Golgi, in addition to further modifications of complex sugars, each gp160 molecule is proteolytically cleaved by cellular proteases into the gp120 surface protein and a gp41 transmembrane subunit [1,2,3]. Env is then transported to the cell surface, where it is incorporated into budding viral particles. Env interaction with CD4 enables coreceptor binding and the subsequent gp41 conformational changes required for a fusion between the virus and the cell membrane [4]. The native unliganded Env is a metastable molecule sampling different conformations. Non-neutralizing antibodies (nnAbs) bind epitopes that are normally occluded in the unliganded trimer and, therefore, fail to recognize the native “closed” trimer, whereas broadly neutralizing antibodies (bNAbs) preferentially recognize this Env conformation [5,6,7,8,9,10]. Clinical trials are currently evaluating the potential of several bNAbs to control viral replication and decrease the size of the viral reservoir [11,12].

In addition to the recent progress made with the development of bNAbs used as prevention and treatment, new inhibitors of HIV-1 are being considered to treat infected individuals. Among them, temsavir (BMS-626529 and GSK2616713), a small molecule entry inhibitor administrated as a prodrug for fostemsavir (BMS-663068, GSK3684934, and RUKOBIA), was recently approved for the treatment of patients who have limited therapeutic options [13,14]. Temsavir is known to bind a conserved pocket in gp120 under its β20–β21 loop, thereby preventing CD4 interaction [15,16]. This molecule also stabilizes the “closed” State 1 Env conformation, which is preferentially recognized by bNAbs [5,7]. 

We and others have reported that temsavir or its BMS-806 analog alter Env glycosylation and cleavage, thereby impacting its recognition by bNAbs [17,18]. Here, we used a panel of primary infectious molecular clones of HIV-1, expression plasmids for wild-type Env, or a cleavage-deficient mutant to evaluate the impact of temsavir on proteolytic Env processing and the recognition of bNAbs. Our findings suggest that the effect of temsavir on Env conformation is linked to its impact on Env cleavage. Albeit to different extents, this effect was observed with multiple Envs and was generally associated with a decreased recognition and antibody-dependent cellular cytotoxicity (ADCC) response mediated by bNAbs. 

## 2. Materials and Methods 

### 2.1. Ethics Statement

Written informed consent was obtained from all study participants, and research adhered to the ethical guidelines of the Centre de recherche du Centre hospitalier de l’Université de Montréal (CRCHUM), which was reviewed and approved by the CRCHUM institutional review board (ethics committee; approval number: CE 16.164–CA; approval date: 19 October 2021). The research adhered to the standards indicated by the Declaration of Helsinki.

### 2.2. Cell Lines and Isolation of Primary Cells

HEK 293T human embryonic kidney cells (obtained from ATCC) were maintained at 37 °C under 5% CO_2_ in Dulbecco’s modified Eagle’s medium (DMEM; Wisent, St. Bruno, QC, Canada) containing 5% fetal bovine serum (FBS; VWR, Radnor, PA, USA) and 100 μg/mL penicillin–streptomycin (Wisent). Primary human peripheral blood mononuclear cells (PBMCs) and CD4^+^ T cells were isolated, activated, and cultured, as previously described [19]. Briefly, PBMCs were obtained by leukapheresis from six HIV-negative individuals (3 males and 3 females). CD4^+^ T cells were purified from rested PBMCs by negative selection (EasySep human CD4^+^ T cell enrichment kit; STEMCELL Technologies, Vancouver, BC, Canada) and were activated with phytohemagglutinin-L (10 µg/mL) for 48 h and then maintained in an RPMI 1640 complete medium supplemented with rIL-2 (100 U/mL).

### 2.3. Plasmids and Proviral Constructs

The vesicular stomatitis virus G (VSV-G)-encoding plasmid was previously described [20]. The sequence of full-length clade B HIV-1_JRFL_ Env and clade A HIV-1_BG505_ (N332) were codon-optimized (GenScript) and cloned into the pcDNA3.1(-) expression plasmid (Invitrogen, Rockford, IL, USA) [21,22]. The plasmids expressing full-length S375W or cleavage-deficient (Cl-) (R508S/R511S) HIV-1_JRFL_ Env were previously described [9]. The R508S/R511S mutations were introduced into the full-length HIV-1_JRFL_ Env S375W by overlapping the PCR to generate the S375W Cl- mutant. The presence of the desired mutations was confirmed by automated DNA sequencing. pSVIIIenv-expressing full-length HIV-1_YU2_ Env and Tat-expressing plasmid pLTR-Tat were previously described [23]. Transmitted/Founder (T/F) and chronic infectious molecular clones (IMCs) of patients CH058, CH077, RHGA, STCO1, and ZM246F-10 were inferred, constructed, and biologically characterized, as previously described [24,25,26,27]. The IMCs encoding for the HIV-1 reference strains JRFL, YU2, and BG505 (T332N) were described elsewhere [28,29,30,31]. Expression plasmids for CH040, RHGA, STCO1, CH198, and ZM246F-10 Env were generated by PCR amplification, inserting the respective Env genes into pCAGGS via EcoRI/XmaI.

### 2.4. Viral Production and Infections

To obtain similar levels of infection in primary CD4^+^ T cells for the different IMCs tested, VSV-G-pseudotyped HIV-1 was produced and titrated as previously described [32]. Viruses were then used to infect activated primary CD4^+^ T cells from healthy HIV-1-negative donors by spin infection at 800× *g* for 1 h in 96-well plates at 25 °C.

### 2.5. Antibodies and HIV+ Plasma

The following Abs were used as primary antibodies for cell-surface Env staining: 10E8, 2G12, N6, VRC01, VRC03, F105, b12, CH106, CD4-Ig, A32, 17b and 19b (NIH AIDS Reagent Program), 3BNC117 (kindly provided by Michel Nussenzweig), F240 (kindly provided by Marzena Pazgier), PGT126, and PGT151 (IAVI). Goat anti-human IgG (H+L) antibodies pre-coupled to Alexa Fluor 647 (Invitrogen) were used as secondary antibodies in the flow cytometry experiments.

### 2.6. Small Molecules

The HIV-1 attachment inhibitor temsavir (BMS-626529) was purchased from APExBIO. The compound was dissolved in dimethyl sulfoxide (DMSO) at a stock concentration of 10 mM and diluted to 10 µM in the RPMI 1640 complete medium for cell-surface staining and ADCC assays for a 24 h treatment.

### 2.7. Radioactive Labeling and Immunoprecipitation of Envelope Glycoproteins

HEK 293T cells (5 × 10^5^) were transfected with an expression plasmid for JRFL Env using the calcium phosphate method. One day post-transfection, cells were metabolically labeled for 16–24 h with 100 μCi/mL of [^35^S] methionine–cysteine ([^35^S] Protein Labeling Mix; PerkinElmer, Waltham, MA, USA) in DMEM lacking methionine and cysteine and supplemented with 10% dialyzed fetal bovine serum and a 1% GlutaMAX™ supplement (Thermo Fisher Scientific, Waltham, MA, USA). Cells were then lysed with a RIPA buffer (140 mM NaCl, 8 mM Na_2_HPO_4_, 2 mM NaH_2_PO_4_, 1% IGEPAL^®^ CA-630 (Sigma-Aldrich, Burlington, MA, USA), 0.05% sodium dodecyl sulfate (SDS), and 1.2 mM sodium deoxycholate). Precipitation of radiolabeled envelope glycoproteins from the whole-cell lysates or found in the supernatant was performed with a pool of plasma from HIV-1-infected individuals in the presence of 45 μL of 10% Protein A-Sepharose beads (Cytiva, Marlborough, MA) at 4 °C. We then loaded the precipitated proteins onto SDS-PAGE gels and analyzed them using autoradiography and densitometry to calculate their processing indices. The processing index was a measure of the conversion of the temsavir-treated gp160 Env precursor to mature gp120 relative to mock-treated Env trimers and took into account the total amount of Env-related bands present in the cell lysate and supernatant. The processing index was calculated with the following formula: processing index = ([total gp120] treated × [gp160] mock-treated)/([gp160] treated × [total gp120] mock-treated). Total referred to gp120 in the cell lysate and supernatant [18,33].

### 2.8. Transfection

Using a standard calcium phosphate method, HEK 293T cells (3 × 10^6^) were co-transfected with 10 μg of a plasmid expressing the gp160 (JRFL WT, JRFL Cl- (R508S/R511S), JRFL S375W, JRFL S375W Cl- (R508S/R511S), CH058, CH077, YU2, BG505, ZM246F-10, STCO1, RHGA, CH040, or CH198) or the pcDNA3.1 plasmid (as a negative control) and 2.5 μg of a green fluorescent protein (GFP) expression plasmid (pIRES2-GFP; Clontech, Mountain View, CA, USA). Cells transfected with the pSVIIIenv YU2 plasmid were co-transfected with 1 μg of a plasmid-encoding pLTR-Tat. Alternatively, cells were transfected with 7 µg of a provirus (CH058 (WT, Cl-) and CH077 (WT, Cl-)) using the calcium phosphate method [33]. The next day, the media were changed for fresh media containing 10 µM temsavir or the equivalent volume of DMSO.

### 2.9. Virus Neutralization Assay

JRFL, CH058, CH077, YU2, BG505, ZM246F-10, STCO1, RHGA, CH040, and CH198 infectious molecular clones of HIV-1 were produced by the calcium phosphate transfection of HEK 293T cells. Two days after transfection, the cell supernatants were harvested. Twenty-four hours before infection, the Cf2Th-T4R5 cells were seeded at a density of 6 × 10^3^ cells/well in 96-well luminometer-compatible tissue culture white plates (Perkin Elmer). Viruses at a final volume of 200 µL were incubated with the indicated temsavir or DMSO dilution (5, 2.5, 1.25, 0.625, 0.3125, 0.156, or 0.078 nM) for 1 h at 37 °C and were then added to the target cells in triplicate, followed by an incubation of 48 h at 37 °C. Cells were then lysed by the addition of 40 µL of a lysis buffer (Promega, Madison, WI, USA), followed by at least 1 h at −80 °C. An LB942 Tri-Star luminometer (Berthold Technologies, Bad Wildbad, Germany) was used to measure the luciferase activity of each well after the addition of 100 mL of a luciferin buffer (15 mM MgSO_4_, 15 mM KPO4 (pH 7.8), 1 mM ATP, and 1 mM dithiothreitol) and 50 mL of 1 mM d-luciferin potassium salt (ThermoFisher Scientific). The neutralization half-maximal inhibitory dilution (IC_50_) represented the temsavir dilution to inhibit 50% of the infection of Cf2Th-T4R5 cells by viruses bearing the indicated surface glycoproteins.

### 2.10. Flow Cytometry Analysis of Cell-Surface Staining

For cell-surface staining, HEK 293T or primary CD4^+^ T cells were incubated for 30 min at 37 °C for 48 h post-transfection/infection with 5 μg/mL of anti-Env monoclonal antibodies or 1:1000 of HIV-1^+^ plasma in PBS. Cells were then washed twice with PBS and stained with 2 μg/mL goat anti-human IgG Alexa Fluor 647 secondary antibodies for 20 min in PBS. After two more PBS washing steps, cells were fixed in a 2% PBS-formaldehyde solution. For the detection of intracellular p24 protein, infected primary CD4^+^ T cells or transfected HEK293T cells were permeabilized using a Cytofix/Cytoperm Fixation/Permeabilization Kit (BD Biosciences, Mississauga, ON, Canada) and intracellularly stained using a PE-conjugated mouse anti-p24 mAb (clone KC57; Beckman Coulter; 1:100 final dilution). The percentage of infected cells (p24+ cells) was determined by gating the living cell population on the basis of viability dye staining (AquaVivid, Thermo Fisher Scientific). Samples were analyzed on an LSRII cytometer (BD Biosciences) and the data analysis was performed using FlowJo v10.5.3 (Tree Star).

### 2.11. FACS-Based ADCC Assay

ADCC was measured with a FACS-based assay 48 h post-infection, as previously described [18,19]. HIV-1-infected primary CD4^+^ T cells were used as target cells and stained with AquaVivid viability dye and cell proliferation dye (eFluor670; eBioscience). Autologous PBMC effector cells, stained with another cellular marker (cell proliferation dye eFluor450; eBioscience, Waltham, MA, USA), were added at an effector:target ratio of 10:1 in 96-well V-bottom plates (Corning, Glendale, AZ, USA). mAbs (5 µg/mL) were added to the appropriate wells and the cells were incubated for 5 min at room temperature. The plates were subsequently centrifuged for 1 min at 300× *g* and incubated at 37 °C and 5% CO_2_ for 5 h before being fixed in a 2% PBS-formaldehyde solution. Cells were then permeabilized using a Cytofix/Cytoperm Fixation/Permeabilization Kit (BD Biosciences) and intracellularly stained using a PE-conjugated mouse anti-p24 mAb (clone KC57; Beckman Coulter; 1:100 final dilution). Samples were acquired on an LSRII cytometer (BD Biosciences) and the data analysis was performed using FlowJo v10.5.3 (Tree Star). ADCC (%) was calculated with the following formula: (% of p24+ cells in targets plus effectors) − (% of p24+ cells in targets plus effectors plus antibody)/(% of p24+ cells in targets) by gating on living target cells.

### 2.12. Detection of Soluble gp120 by Sandwich ELISA

The concentration of soluble gp120 in the supernatant of gp160-expressing cells was determined by an anti-gp120 sandwich ELISA. A combination of nnAbs 17b and A32 was prepared in PBS and adsorbed to plates (MaxiSorp; Nunc) overnight at 4 °C. The coated wells were subsequently blocked with a blocking buffer (Tris-buffered saline (TBS) containing 0.1% Tween 20 and 2% BSA) for 90 min at room temperature. The wells were then washed four times with a washing buffer (Tris-buffered saline (TBS) containing 0.1% Tween 20). Supernatants from gp160-expressing cells were incubated with the nnAbs-coated wells for 120 min at room temperature. The plates were washed four times with a washing buffer, followed by incubation with HRP-conjugated C11 nnAbs (3 mg/mL) for 90 min at room temperature. HRP enzyme activity was determined after the addition of a 1:1 mix of Western Lightning oxidizing and luminol reagents (Perkin Elmer Life Sciences, Waltham, MA, USA). Light emission was measured with an LB942 Tri-Star luminometer (Berthold Technologies, Bad Wildbad, Germany). A standard curve using known concentrations of purified recombinant gp120YU2 was used to quantify the precise concentration of soluble gp120 in each supernatant.

### 2.13. Statistical Analysis

Statistics were analyzed using GraphPad Prism version 9.1.0 (GraphPad). Every dataset was tested for statistical normality and this information was used to apply the appropriate (parametric or non-parametric) statistical test based on statistical normality. *p* values < 0.05 were considered to be significant; significance values were indicated as * *p* < 0.05, ** *p* < 0.01, *** *p* < 0.001, **** *p* < 0.0001.

## 3. Results

### 3.1. The Effect of Temsavir on Env Depends on Proteolytic Env Processing

**Figure 1:**
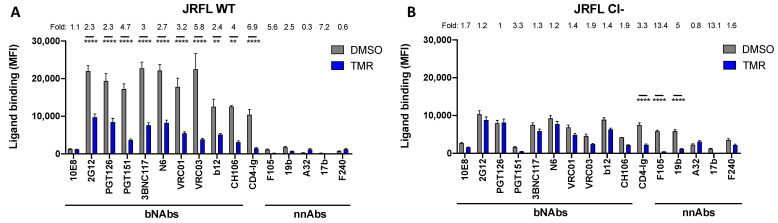
**Env cleavage affects the capacity of temsavir to modulate bNAb recognition.** HEK 293T cells were transfected with a plasmid expressing the (**A**) HIV-1_JRFL_ Env WT or (**B**) its cleavage-deficient counterpart (Cl-), together with a plasmid-expressing GFP. Cells were then treated with 10 µM temsavir (TMR) or the equivalent volume of DMSO for 24 h. Cell-surface staining was performed using a panel of bNAbs (10E8, 2G12, PGT126, PGT151, 3BNC117, N6, VRC01, VRC03, b12, and CH106), nnAbs (F105, 19b, A32, 17b, and F240), and CD4-Ig. Shown are mean fluorescence intensities (MFI) ± standard error of the mean (SEM). MFI values were determined on the transfected (GFP+) population. The data shown represent results obtained from at least four independent experiments per ligand. Statistical significance was tested using a two-way analysis of variance (ANOVA) (** *p* < 0.01; **** *p* < 0.0001).

We recently reported that the treatment of Env-expressing cells with temsavir for 24 h resulted in a decrease in the proteolytic processing and bNAbs recognition of HIV-1_JRFL_ Env [18]. To better characterize the role of Env cleavage in bNAbs recognition, we evaluated the impact of temsavir on a gp160 cleavage-deficient (Cl-) Env mutant. In this mutant, the two conserved arginine residues at positions 508 and 511 were replaced by two serine residues. Mutations of the primary cleavage site were shown to almost completely abrogate the JRFL Env cleavage [34]. To confirm the impact of these mutations on Env processing, we used the cleavage-dependent PGT151 bNAb as a control. As expected, PGT151 recognized Cl- Env less efficiently than its wild-type counterpart (Figure 1 and Figures S1 and S2). Of note, previous work has shown that nnAbs do not bind the unliganded “closed” Env trimer [9,33,35], which is stabilized by Env cleavage [17,36,37,38]. A mutation of the furin cleavage site results in the spontaneous sampling of downstream “open” conformations, thus enabling recognition by nnAbs [9,17,39]. We, therefore, evaluated the effect of temsavir on bNAbs recognition. Env-expressing cells (Figure 1 and Figure S1) and infected primary CD4^+^ T cells (Figure S2) were treated (or not) with temsavir for 24 h before measuring the binding of bNAbs by flow cytometry. Although the temsavir treatment significantly reduced the detection of wild-type Env by bNAbs at the cell surface, no major differences were observed for its cleavage-deficient counterpart. As controls, we included CD4 binding site mAbs (3BNC117, N6, VRC01, VRC03, b12, and CH106) and ligands (CD4-Ig), and observed that temsavir competed with them, with the most profound effect detected in CD4-Ig (Figure 1). Similar results were obtained with the CH058 and CH077 Cl- strains (Figures S1 and S2). Finally, the effect of temsavir was specific because no effect was observed with the temsavir-resistant Env S375W mutant (Figure S3) [18]. The presence of tryptophan residue instead of a serine filled the cavity and, therefore, prevented temsavir from binding [40].

### 3.2. Temsavir Decreases Glycosylation and the Proteolytic Cleavage of Env, Resulting in Decreased Recognition by bNAbs

**Figure 2:**
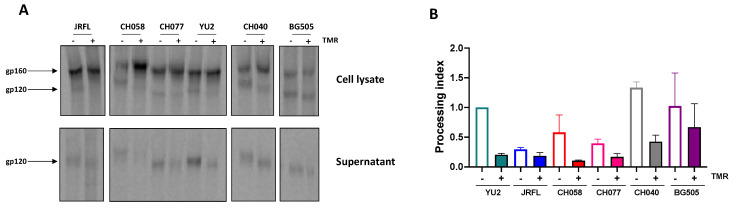
**Temsavir modifies glycosylation and processing of HIV-1 Env.** (**A**) HEK 293T cells were transfected with a plasmid expressing HIV-1 Env (JRFL, CH058, CH077, YU2, CH040, and BG505) and labeled for 24 h with [^35^S] methionine and [^35^S] cysteine. Cells were then treated with 10 µM temsavir (TMR) or the equivalent volume of DMSO. Cell lysates and supernatants were immunoprecipitated with plasma from HIV-1-infected individuals. The precipitated proteins were loaded on SDS-PAGE gels and analyzed by autoradiography. (**B**) Quantification of the effect of temsavir on Env processing normalized to YU2 in the presence of the vehicle (DMSO).

As Env cleavage appeared to be important for the effect of temsavir on recognition by some bNAbs, we investigated the impact of temsavir on the processing of Env from different HIV-1 strains, including transmitted/founder and chronic viruses. Briefly, we transfected HEK 293T cells with plasmids encoding Env from HIV-1 clades A (BG505), B (JRFL, CH058, CH077, YU2, CH040, RHGA, and STCO1), or C (ZM246F and CH198). Cells were treated (or not) with temsavir for 24 h before measuring Env processing by radioactive labelling, followed by immunoprecipitation. The treatment of Env-expressing cells with temsavir decreased the cleavage of most of the Envs tested (Figure 2 and Figure S4), thereby confirming the broad impact of temsavir on Env processing. Of note, we observed that upon temsavir treatment, gp120 migrated faster for the different Envs tested, particularly evident in the supernatant (Figure 2A). This accelerated migration pattern was consistent with the effect of temsavir on glycosylation. In addition, the levels of shed gp120 in the supernatant decreased upon temsavir treatment, as previously shown [41].

**Figure 3:**
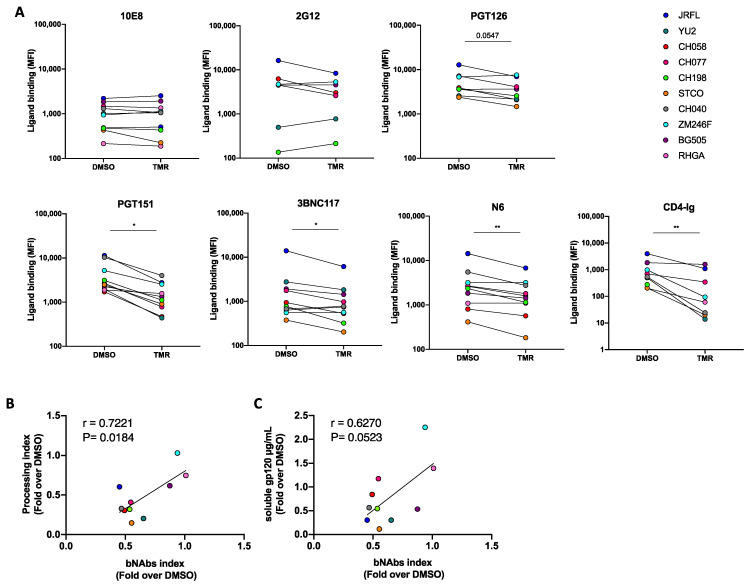
**Temsavir treatment affects Env recognition by bNAbs.** (**A**) HEK 293T cells were transfected with plasmid-expressing HIV-1 clade A (BG505), B (JRFL, YU2, CH058, CH077, CH040, RHGA, and STCO1), or C (ZM246F and CH198) Envs, together with a plasmid-expressing GFP. Cells were then treated with 10 µM temsavir (TMR) or the equivalent volume of DMSO for 24 h. Cell-surface staining was performed using a panel of bNAbs (10E8, 2G12, PGT126, PGT151, 3BNC117, and N6) and the ligand CD4-Ig 24 h post-treatment and 48 h post-transfection. Shown are the mean fluorescence intensities (MFI) using the different ligands. MFI values were measured on the transfected (GFP+) population. The data shown represent results obtained from at least five independent experiments. (**B**) Correlation of the Env processing index and bNAbs (2G12, PGT126, PGT151, 3BNC117, and N6) index. (**C**) Correlation of the soluble gp120 index and bNAbs (2G12, PGT126, PGT151, 3BNC117, and N6) index. (**B**,**C**) are represented as fold TMR over DMSO for each Env tested. Statistical significance was tested using (**A**) a paired *t*-test or Wilcoxon signed-rank test based on statistical normality (* *p* < 0.05; ** *p* < 0.01) or (**B**,**C**) a Pearson correlation test.

To determine if the changes in Env processing and glycosylation described above affected Env conformation, we analyzed Env recognition with a panel of bNAbs. Briefly, HEK 293T cells transfected with plasmids encoding Env from HIV-1 clades A, B, and C were treated with temsavir, or the vehicle alone (DMSO), 24 h post-transfection. The binding of bNAbs was evaluated by flow cytometry 48 h post-transfection. The most striking phenotype observed was a significant decrease in PGT151 recognition upon temsavir treatment for all Envs tested (Figure 3A). As expected, from competition for the CD4 binding site, CD4BS antibodies (3BNC117 and N6) and CD4-Ig bound cell-surface Env less efficiently upon temsavir treatment. Of note, we observed a positive correlation between a temsavir-mediated decrease in Env processing and bNAbs (2G12, PGT126, PGT151, 3BNC117, and N6) recognition (Figure 3B), suggesting that bNAbs interaction in the presence of temsavir is affected by Env cleavage. Accordingly, the effect of temsavir on bNAbs recognition was also associated, albeit not statistically significantly, with its effect on gp120 shedding (Figure 3C).

### 3.3. Temsavir Affects Env Recognition of Infected Primary CD4^+^ T Cells

**Figure 4:**
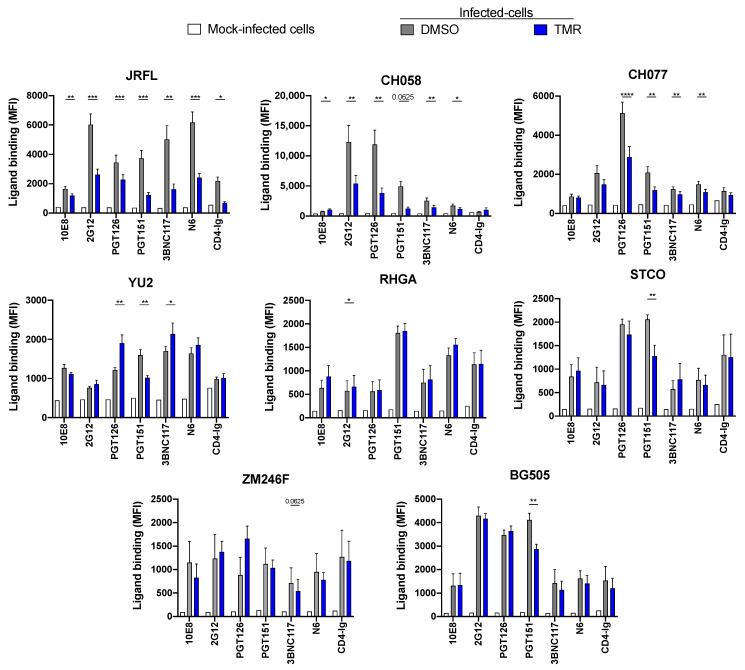
**Temsavir affects the recognition of HIV-1-infected primary CD4^+^ T cells by bNAbs.** Primary CD4^+^ T cells were infected with HIV-1 clade A (BG505), B (JRFL, YU2, CH058, CH077, RHGA, and STCO), and C (ZM246F). Cells were then treated with 10 µM temsavir (TMR) or the equivalent volume of DMSO for 24 h. Cell-surface staining was performed using a panel of bNAbs (10E8, 2G12, PGT126, PGT151, 3BNC117, and N6) and the ligand CD4-Ig. Shown are the mean fluorescence intensities (MFI) using the different ligands ± standard error of the mean (SEM). MFI values were measured from the infected (p24^+^) population. The data shown represent results obtained from at least five independent experiments per ligand. Statistical significance was tested using a paired *t*-test (* *p* < 0.05; ** *p* < 0.01; *** *p* < 0.001; **** *p* < 0.0001).

To evaluate whether our results could be translated to the recognition of infected primary CD4^+^ T cells, we produced infectious viral particles from the corresponding infectious molecular clones. Primary CD4^+^ T cells were then infected with these viruses and treated (or not) with temsavir for 24 h before measuring the binding of bNAbs by flow cytometry, as previously described [18]. Again, we observed a significant decrease in the recognition of HIV-1-infected cells by PGT151, which was consistent with decreased Env processing. This phenotype was observed with most IMCs, with the exception of RHGA and ZM246F, for which temsavir had a minimal impact on Env cleavage (Figure 3B and Figure S4). We observed a differential impact of temsavir treatment on Env recognition among the different strains with the other bNAbs (Figure 4). Although all these Envs were susceptible to temsavir-mediated viral inhibition, as measured in a single-round pseudoviral neutralization assay, their susceptibility to temsavir varied (Table S1).

### 3.4. Impact of Temsavir on bNAb Recognition Affects ADCC Responses

**Figure 5:**
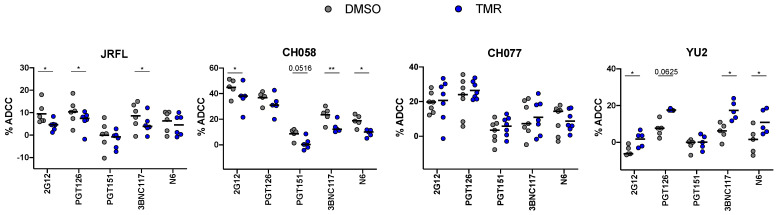
**Impact of temsavir on ADCC responses mediated by selected bNAbs.** Primary CD4^+^ T cells were infected with HIV-1 JRFL, YU2, CH058, and CH077. Cells were then treated with 10 µM temsavir (TMR) or the equivalent volume of DMSO for 24 h. These primary infected cells were used as target cells and co-cultured with autologous peripheral blood mononuclear cells (PBMCs) as effector cells in a FACS-based ADCC assay using 2G12, PGT126, PGT151, 3BNC117, and N6. The graphs shown represent the mean percentage of ADCC obtained for each antibody after DMSO or TMR treatment. The data shown represent results obtained from at least five independent experiments. Statistical significance was tested using a paired *t*-test (* *p* < 0.05; ** *p* < 0.01).

To evaluate whether the differences in Env recognition at the surface of the infected cells translated into a functional consequence, we measured their capacity to mediate ADCC. Indeed, the decreased recognition of JRFL- and CH058-infected cells by 2G12, PGT126, PGT151, 3BNC117, and N6 mAbs (Figure 4) translated into decreased ADCC responses (Figure 5). Of note, we observed no differences in ADCC for CH077 (Figure 5). Interestingly, for YU2-infected cells, we observed an increase in ADCC, mediated by 2G12, PGT126, 3BNC117, and N6 (Figure 5), which was consistent with an improved recognition by these bNAbs (Figure 4). These seemingly counterintuitive results could be related to the capacity of temsavir to reduce YU2 gp120 shedding and, therefore, enhance the level of cell-surface Envs (Figure 2, Figure 3 and Figure 4), as previously reported [41]. Although the mechanisms explaining these phenotypes are not completely clear at the moment, the pleiotropic effect of temsavir on processing, glycosylation, shedding, and Env conformation might account for this. Another possibility that could have contributed to this phenotype is the potential effect of temsavir treatment on Env expression at the cell surface. However, we did not observe any effect of temsavir treatment on 10E8 binding (Figure 1, Figure 3 and Figure 4), suggesting that Env cell-surface expression was not the main driver of this effect. Further work to identify the mechanisms behind the heterogeneity of temsavir treatment on Env conformation is therefore warranted.

## 4. Discussion

Strategies aiming at the elimination of HIV-1-infected cells are the focus of intense research. Env expression at the surface of infected cells makes it a target of choice for antibody-based interventions, thus the characterization of the Env conformational landscape remains an important topic of research. Multiple factors can influence Env conformation such as accessory proteins, Env proteolytic cleavage, and small molecules inhibitors [35,42]. The small molecule inhibitor temsavir is an attachment inhibitor reported to stabilize the pre-triggered (State 1) “closed” conformation. Likely resulting from the intracellular stabilization of the “closed” conformation, the addition of temsavir to Env-expressing cells affects glycosylation and proteolytic cleavage [17,18]. Here, we extended these observations to additional primary Envs, and explored the effect of temsavir on Env cleavage as the underlying mechanism for its impact on bNAb recognition. Accordingly, a cleavage-deficient Env was resistant to temsavir, as highlighted by an absence of a decrease in recognition by bNAbs, whereas recognition by the CD4-Ig ligand was decreased, likely due to competition with the small molecule. This was consistent with recent structural studies revealing that temsavir engages gp120 in a pocket under the β20–β21 loop, sequestering three key CD4 contact residues, N425, M426, and W427 [15,16]. Altogether, these results suggest that temsavir mostly modifies Env conformation by altering its cleavage. Although we could not rule out a potential impact of temsavir on cell-surface Env expression, we noted that temsavir did not affect the binding of all bNAbs to the same extent. Future studies are needed to better characterize the mechanism by which temsavir affects Env conformation.

Supporting a conserved mode-of-action for different HIV-1 Envs, we observed a decrease in proteolytic processing for most of the Envs tested. The effect of temsavir on cleavage and, therefore, Env conformation was reflected in a decreased recognition of most bNAbs tested. We observed, however, some heterogeneity related to this phenotype. This heterogeneity could not be totally explained by the neutralization sensitivity of the different Env pseudotype particles to temsavir (Table S1). However, we observed a variability in the effect of temsavir on intrinsic Env properties such as processing, glycosylation, and shedding. Therefore, the susceptibility of the different Envs to the pleiotropic effects of temsavir likely explains why some Envs are more sensitive (JRFL, CH058, CH077, CH040, and CH198) than others (RHGA, STCO1, ZM246F, BG505, and YU2) to temsavir treatment.

We noted that the effect of temsavir on Env expressed at the surface of primary CD4^+^ T cells showed differences in some instances to what was seen in CD4-negative Env-expressing cells. We believe that several factors likely explain these differences, including differential gp160 processing among these cell types and the presence of the CD4 receptor. Of note, we observed a consistent and significant decrease in Env recognition by the cleavage-sensitive PGT151 bNAb for most Envs. When a decrease in bNAbs recognition was observed at the surface of infected primary CD4^+^ T cells, this was usually translated into a decreased ADCC. Temsavir is a potent attachment inhibitor, but given its impact on Env processing, glycosylation, and conformation, we believe that these additional activities need to be considered for the development of future immunotherapies combining temsavir with antibody-based treatments.

## Data Availability

Further information, data reported in this paper, and requests for resources and reagents should be directed to and will be fulfilled by the lead contact, Andrés Finzi (andres.finzi@umontreal.ca) upon request.

## Supplementary Materials

The following supporting information can be downloaded at https://www.mdpi.com/article/10.3390/v15051189/s1. Figure S1: Env cleavage affects the capacity of temsavir to modulate bNAb recognition on CH058 and CH077 strains at the surface of HEK 293T cells. Figure S2: Env cleavage affects the capacity of temsavir to modulate bNAb recognition on CH058 and CH077 strains at the surface of primary CD4+ T cells. Figure S3: Temsavir treatment does not affect ligand recognition for the resistant mutants Env_S375W_ and Env_S375W Cl-_. Figure S4: Effect of temsavir on Env cleavage. Table S1: Temsavir neutralization half-maximal inhibitory concentration (IC_50_) of multiple HIV-1 strains.
