# Supplementary material for: Temsavir Modulates HIV-1 Envelope Conformation by Decreasing Its Proteolytic Cleavage
Source: Viruses. 2023 May 18;15(5):1189. doi: 10.3390/v15051189 (PMC10221371; doi:10.3390/v15051189)
Supplement: Supplementary file 1 [file viruses-15-01189-s001.zip › viruses-2340918-supplementary.pdf]

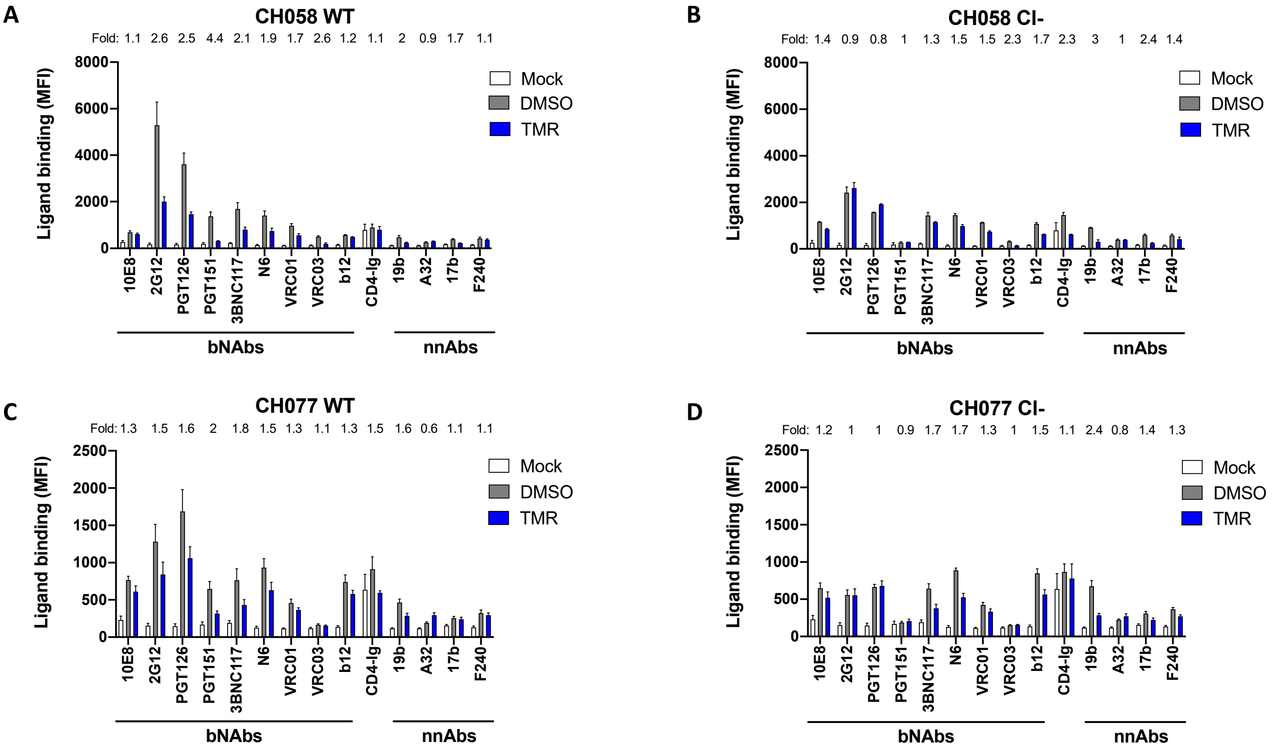

Figure S1: Env cleavage affects the capacity of temsavir to modulate bNAb recognition of CH058 and CH077 Envs at the surface of HEK 293T cells. HEK 293T cells were transfected with primary IMCs (A) CH058 WT, (B) CH058 Cl-, (C) CH077 WT, and (D) CH077 Cl-. Cells were then treated with 10 µM temsavir (TMR) or the equivalent volume of DMSO for 24 h. Cell surface staining was performed using a panel of bNAbs (10E8, 2G12, PGT126, PGT151, 3BNC117, N6, VRC01, VRC03, and b12), nnAbs (19b, A32, 17b, and F240), and CD4-Ig. Shown are mean fluorescence intensities (MFI) ± standard error of the mean (SEM). MFI values were determined on the transfected (p24+) population. The data shown represents results obtained from at least 2 independent experiments per ligand.

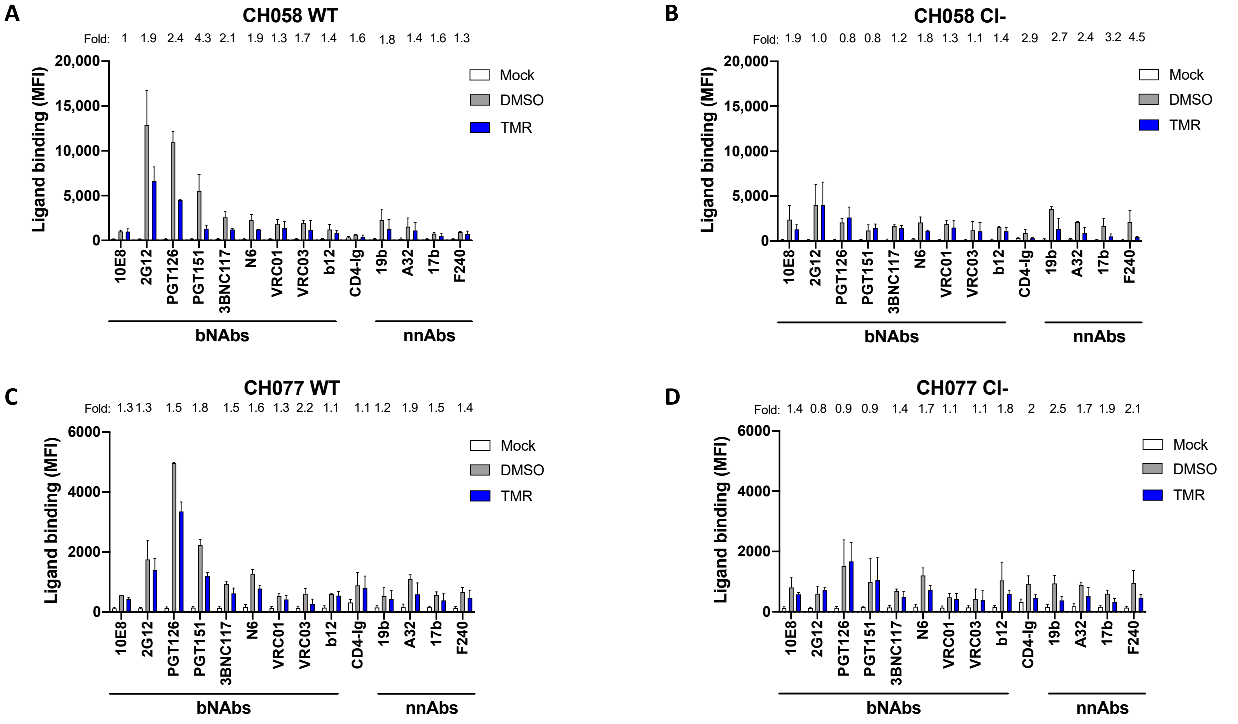

Figure S2: Env cleavage affects the capacity of temsavir to modulate bNAb recognition of CH058 and CH077 Envs at the surface of primary CD4$^+$ T cells. Primary CD4$^+$ T cells were infected with (A) CH058 WT, (B) CH058 Cl-, (C) CH077 WT, and (D) CH077 Cl- viruses. Cells were then treated with 10 µM temsavir (TMR) or the equivalent volume of DMSO for 24 h. Cell surface staining was performed using a panel of bNAbs (10E8, 2G12, PGT126, PGT151, 3BNC117, N6, VRC01, VRC03, and b12), nnAbs (19b, A32, 17b, and F240), and CD4-Ig. Shown are mean fluorescence intensities (MFI) ± standard error of the mean (SEM). MFI values were determined on the transfected (p24+) population. The data shown represents results obtained from at least 2 independent experiments per ligand.

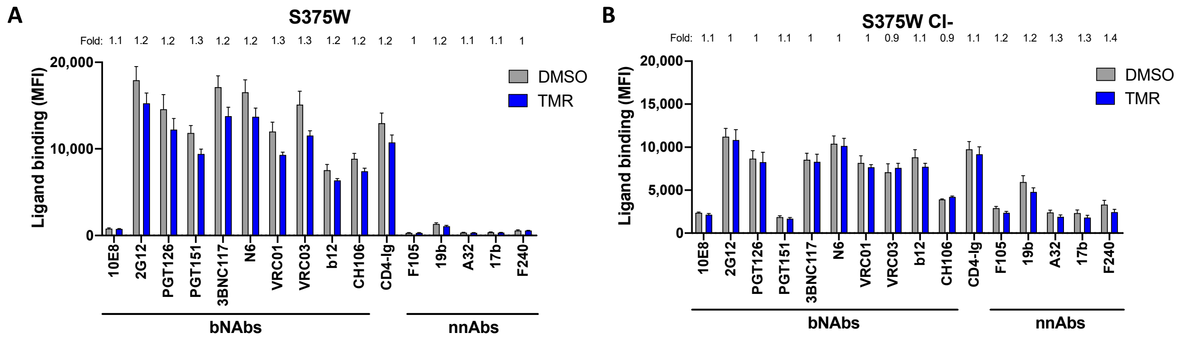

Figure S3: Temsavir treatment does not affect ligand recognition for the resistant-mutants Env$_{S375W}$ and Env$_{S375W\ Cl-}$.

HEK 293T cells were transfected with a plasmid expressing (**A**) HIV-1$_{JR-FL}$ Env S375W or (**B**) HIV-1$_{JR-FL}$ Env S375W Cl-, together with a plasmid expressing GFP. Cells were then treated with 10 µM temsavir (TMR) or the equivalent volume of DMSO for 24 h. Cell surface staining was performed using a panel of bNAbs (10E8, 2G12, PGT126, PGT151, 3BNC117, N6, VRC01, VRC03, b12, and CH106), nnAbs (F105, 19b, A32, 17b, and F240), and the ligand CD4-Ig. Shown are mean fluorescence intensities (MFI)± standard error of the mean (SEM). MFI values were measured on the transfected (GFP+) population. The data shown represents results obtained from at least four independent experiments for each ligand. Statistical significance was tested using a two-way analysis of variance (ANOVA).

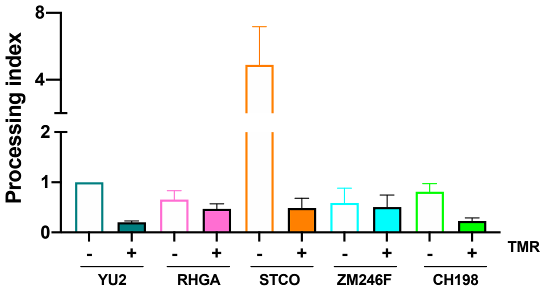

Figure S4: Effect of temsavir on Env cleavage.

HEK 293T cells were transfected with plasmids expressing Env (YU2, RHGA, STCO, ZM246F, and CH198) and labeled for 24 h with [^{35}S] methionine and [^{35}S] cysteine in the presence of 10 µM temsavir or the equivalent volume of DMSO. Cell lysates and supernatants were immunoprecipitated with plasma from HIV-1-infected individuals. The precipitated proteins were load on SDS-PAGE gels and analyzed by autoradiography. The effect of temsavir on Env processing was quantified and normalized to YU2 in the presence vehicle (DMSO)

Env	Neutralization IC_{50} (nM)
YU2	0,22
JRFL	0,16
CH058	0,22
CH077	0,13
CH198	0,08
STCO	1,30
CH040	0,18
ZM246F	0,08
BG505	6,40
RHGA	0,18

Table S1: Half-maximal inhibitory concentrations (IC_{50}) of multiples HIV-1 strains by temsavir. Pseudoviral particles coding for the luciferase reporter gene and bearing the following glycoproteins: JRFL, CH058, CH077, YU2, CH040, RHGA, BG505, STCO, ZM246F, and CH198 were used to infect Cf2Th-T4R5 cells. Pseudoviruses were incubated with increasing concentrations of temsavir for 1 h at 37°C prior infection. Data represents the average from at least three independent experiments.
